# Short Notices of Hospital Therapeutics

**Published:** 1854-07

**Authors:** 


SHORT NOTICES OF HOSPITAL THERAPEUTICS.

*To Prevent Night-sweats in Phthisis.*—Night-perspirations in the course of phthisis are often extremely annoying to the patient; they appear, also, to be simply debilitating, and unattended by any degree of collateral benefit. Some cases which were carefully noted by Mr. Hutchinson, the Clinical Assistant at the City Hospital for Chest Diseases, with a view to the determination of that question, appeared to show that they may be artificially checked, not only with impunity, but with great benefit. The patients who were so treated, and who, in the course of a week or a fortnight, got quite rid of sweatings which for months had been profuse, thought themselves much better, and did not complain of increase, either as regards the expectoration, the feverishness, or the sense of stuffing in the chest. Under the usual treatment of phthisis, (full diet, cod-liver oil, and tonics,) the tendency to night-perspiration often ceases spontaneously. If it becomes desirable to expedite the process, it may be done by the sesquichloride of iron, the mineral acids, or, best of all, by the gallic acid. The following is the prescription for a night-draught containing the latter.—

℞ Acidi gallici. gr. vij.; morph. acet. gr. ⅛; alcohol q. s. (a few drops); syr. tolutan. ʒss.; aquæ ℥j.

The night-pill, as we find in the Pharmacopœia of the Brompton Hospital for Consumption, is—

℞ Acid. gallic. gr. v.; morph. hydrochl. gr. ⅛; mist. acac. q. s. Ft. pil. ij.

It is also of advantage to adopt an astringent regimen as far as convenient. The patient should be directed to sleep on a mattress, alone, and not heavily clothed; he should wear no flannel in bed; as dry a diet should be taken as conveniently can be borne, and fluid should be especially avoided in the latter half of the day, none whatever being allowed later than several hours before bed-time.

*Treatment of Milk Abscess.*—We alluded in terms of strong commendation, in a former report, to the plan of tonic medication in all cases of milk abscess, which is adopted by Mr. Paget at St. Bartholomew's Hospital. Very numerous cases of this affection, in all forms and stages, excepting perhaps the peracute, come under treatment in the out-patients' rooms of that Institution. Their subjects are generally cachectic women. Mr. Paget always opens the abscess as soon as fluctuation can be felt, by an incision as small as possible, scrupulously abstains from squeezing, and orders a small poultice to be applied over the part where the skin is inflamed. Over the poultice a layer of cotton-wool is placed, and the whole slung to the neck in a handherchief. A draught, containing quinine and the sulphate of iron, is ordered to be taken three times daily. Mr. Paget states, that, for many years, he has never, in a single case, had any trouble with fistulæ. We observe a great difference in practice among Hospital Surgeons as to making large or small incisions into mammary abscesses; some preferring very free openings, and others, like Mr. Paget, never making more than a mere puncture. On the whole, the latter mode appears to be the more successful; the constitutional treatment is doubtless, however, of the most importance.

*Remedy for Favus.*—From the observation of about a dozen cases of severe favus (diagnosis by the microscope in all) recently treated by Mr. Startin at the Hospital for Skin Diseases, we can speak with great confidence of the efficiency of the following ointment. It is the Ung. sulph. comp. of the Pharmacopœia of that Institution. ℞ Sulph. sublimati lbss.; hydrarg. ammoniochloride. ℥ss.; hydr. sulphureti cum sulph. ℥ss. Leviga simul, dein adde olivæ olei ℥iv.; adipis recentis ℥xvj.; creosoti ♏xx.; misce. To correct the state of general health, Mr. Startin commonly orders simultaneously a mild course of the iodide of potassium, but this, we suspect, has but a small share, if any, in the local result. Often when the scalp has been for many years thickly covered with the peculiar favus crust, four or five nightly applications of the above ointment have sufficed to make it perfectly clean. So long as the patient will continue regularly to use a small quantity every day, the disease may be prevented from reappearing, and the condition assumed by the scalp under its influence might easily be mistaken by the inexperienced for one of complete cure. As soon, however, as the inunction is suspended, the eruption reappears. This liability we have known, in more than one case, to extend over nearly a year, and probably it may for much longer periods. The ointment, however, which does not smell much, need only be applied at night, and may be washed entirely away every morning, so as to entail but little inconvenicnce on the patient. The hair will, to a considerable extent, grow during the treatment, provided that the scalp have not been too much destroyed. In a most disgusting disease, for which as yet no real cure is known, it is much to be in possession of an almost certain means of ensuring its absence. The ointment no doubt acts as a parasiticide. Before its first application it is desirable to clear away the crust as much as possible, either by fomentation or a poultice.

We may remark, that the ointment mentioned is used by Mr. Startin in the treatment of scabies, and also in that of the contagious form of porrigo.

*Dilute Nitric Acid in Constitutional Syphilis.*—A practical rule of great value in the management of the constitutional forms of syphilitic disease is, never to persevere with a remedy which, after a fair trial, produces no apparent benefit. It is a disease which, if the right treatment be hit upon, generally yields with surprising rapidity. Another rule, which we observe most practical surgeons adopt, is, to change the remedy, if, after great improvement, the affection come to a stand-still or appear to retrograde. By ringing changes on the iodide of potassium, small doses of the bichloride, iodide, or biniodide of mercury, or the dilute nitric acid, much greater good may be done, than by the continuous employment of any one of these preparations. The dilute nitric acid is a drug which has a considerable reputation in this disease, but of which the exact effects have not been sufficiently investigated. Whether it acts merely as a tonic or alterative, or whether it has specific power over the disease, is yet a debated question. The following case, in which, after the iodide had failed, it produced effects which might seem to denote a specific influence, is worthy of narration. We epitomize it from the daily record kept by Mr. Edmonds, the dresser of the patients:

E. J., aged 20, a prostitute, was admitted into St. Thomas's, under Mr. Simon's care, on April 26, suffering severely from an eruption of ulcerating rupia, the large sores formed by which were scattered over the thighs, nates, and some other parts of the body. The disease had marked syphilitic characters, and she admitted having been salivated for the original affection one year previously. Mr. Simon ordered a meat diet, with eight ounces of wine daily; the sores to be dressed with a bichloride lotion (gr. ij. ad ℥j.), and a draught containing four grains of iodide of potassium dissolved in water, to be taken three times daily. These measures were continued for ten days without benefit, the patient meanwhile taking her food well. On May 6, some of the sores were decidedly increasing, and one of the larger was in a very sloughy state. The draught was now changed for ℞ Acid. nitric. dilut. ♏xxv., syrupi ʒij., quin. disulph. gr. ii., aquæ ℥j. The same lotion was continued, the quantity of wine increased to twelve ounces, and two eggs added to the daily allowance of food. A surprisingly rapid improvement ensued on this change of measures. The sores at once cleaned and commenced to heal. In the course of ten days several were almost cicatrized, and the patient is now so nearly well that she will shortly be discharged. It is possible, of course, that the quinine in this case, which was commenced simultaneously with the acid, had some share in the cure. As, however, a tonic regimen, with wine, had been pursued before, without the least benefit, it seems fair to attribute the chief influence to the latter.—*Lon. Med. Times.*

